# Binding of Regulatory Subunits of Cyclic AMP-Dependent Protein Kinase to Cyclic CMP Agarose

**DOI:** 10.1371/journal.pone.0039848

**Published:** 2012-07-09

**Authors:** Andreas Hammerschmidt, Bijon Chatterji, Johannes Zeiser, Anke Schröder, Hans-Gottfried Genieser, Andreas Pich, Volkhard Kaever, Frank Schwede, Sabine Wolter, Roland Seifert

**Affiliations:** 1 Institute of Pharmacology, Hannover Medical School, Hannover, Germany; 2 Institute of Toxicology, Hannover Medical School, Hannover, Germany; 3 Biolog Life Science Institute, Bremen, Germany; Griffith University, Australia

## Abstract

The bacterial adenylyl cyclase toxins CyaA from *Bordetella pertussis* and edema factor from *Bacillus anthracis* as well as soluble guanylyl cyclase α_1_β_1_ synthesize the cyclic pyrimidine nucleotide cCMP. These data raise the question to which effector proteins cCMP binds. Recently, we reported that cCMP activates the regulatory subunits RIα and RIIα of cAMP-dependent protein kinase. In this study, we used two cCMP agarose matrices as novel tools in combination with immunoblotting and mass spectrometry to identify cCMP-binding proteins. In agreement with our functional data, RIα and RIIα were identified as cCMP-binding proteins. These data corroborate the notion that cAMP-dependent protein kinase may serve as a cCMP target.

## Introduction

Previous studies claimed that in addition to adenosine 3′,5′-cyclic monophosphate (cAMP) and (cytidine 3′,5′-cyclic monophosphate) cGMP [1], [2], the cyclic pyrimidine nucleotide cytidine 3′,5′-cyclic monophosphate (cCMP) may play a role as second messenger molecule [3]. However, studies on cellular effects of cCMP were not reproducible [4] and technical problems hampered the determination of tentative cytidylyl cyclase activity in mammalian cells [5], [6]. Moreover, a postulated cCMP-specific phosphodiesterase could not be identified so far [7]. In fact, several known phosphodiesterases do not cleave cCMP [8]. With refined radiometric and liquid chromatography- mass spectrometry (LC-MS)-based methods we could recently show that the highly purified bacterial adenylyl cyclase toxins CyaA from *Bordetella pertussis* and edema factor from *Bacillus anthracis*, in addition to cAMP, produce cCMP [9]. Furthermore, the highly purified soluble guanylyl cyclase α_1_β_1_ along with cGMP, produces cCMP in a nitric oxide-dependent manner [10]. In addition, the regulatory subunits of cAMP-dependent protein kinase A (PKA), RIα and RIIα, are activated not only by cAMP, but by cCMP as well [11]. These recent data indicate that cCMP may, indeed, play a role as second messenger.

**Figure 1:**
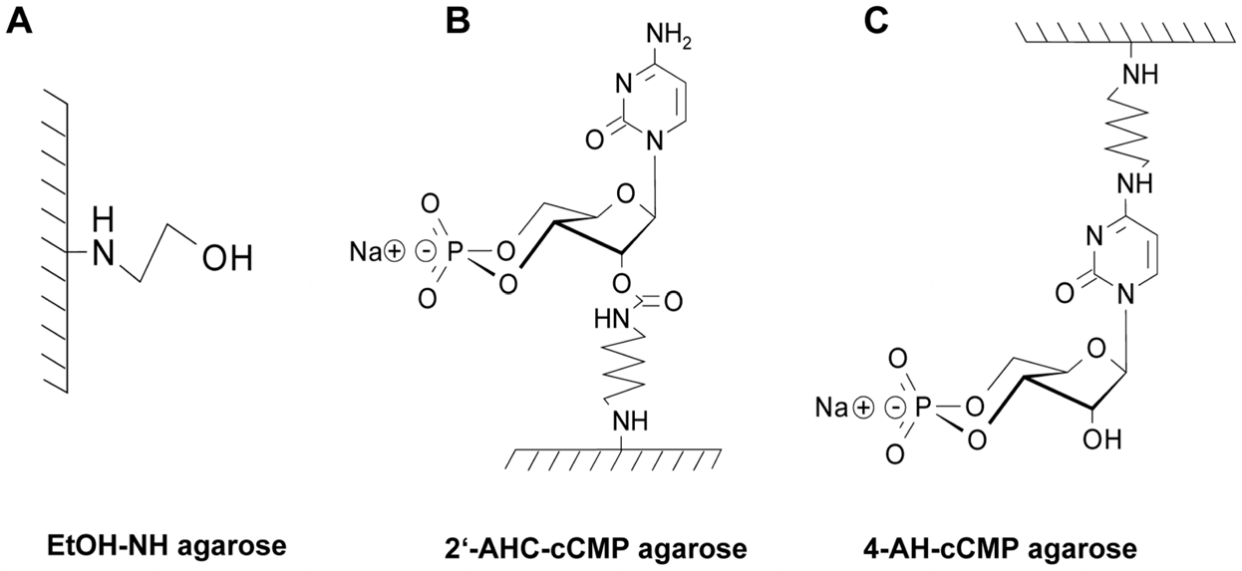
Structures of agarose matrices. A, EtOH-NH agarose (control agarose); B, 2′-AHC-cCMP agarose; C, 4-AH-cCMP agarose. The matrices shown in this figure were used as novel tools for identification of cCMP-binding proteins. Please, note the different attachments of the affinity ligand to the matrix in B and C.

The aim of our present study was to identify cCMP-binding proteins. As methodological approach, we synthesized and tested 2′-6-aminohexylcarbamoyl-cCMP (2′-AHC-cCMP) agarose and 4-6-aminohexyl-cCMP (4-AH-cCMP) agarose and a corresponding control agarose (Figure 1). In 2′-AHC-cCMP agarose, the nucleoside 3′,5′-cyclic monophosphate (cNMP) is linked to the matrix via the 2′-O-ribosyl group, and in 4-AH-cCMP agarose via the 4-NH group of the pyrimidine ring. Hence accessibility of the affinity ligand to proteins is different in the two matrices. Bound proteins were subsequently analyzed by immunoblotting and LC-MS. The cNMP-agarose approach is very useful at identifying cNMP-binding proteins [12]. Here, we show that in accordance with our enzymological data, cCMP-agarose binds RIα and RIIα.

## Materials and Methods

### Materials

2′-AHC-cCMP agarose was synthesized by analogy to other 2′-AHC-agarose matrices [13]. Syntheses of 4-AH-cCMP and 4-AH-cCMP agarose were in accordance to literature procedures [14], [15]. Both cCMP agaroses were prepared with ligand densities of ∼6 µMol/mL of settled gel. cCMP (purity > 99,8%) was from Biolog Life Science Institute (Bremen, Germany). Anti-RIα Ig (sc-136231) was purchased from Santa Cruz Biotechnology (Santa Cruz, CA, USA). This antibody also recognizes RIβ. All other reagents and cell culture media were purchased from standard suppliers.

**Figure 2:**
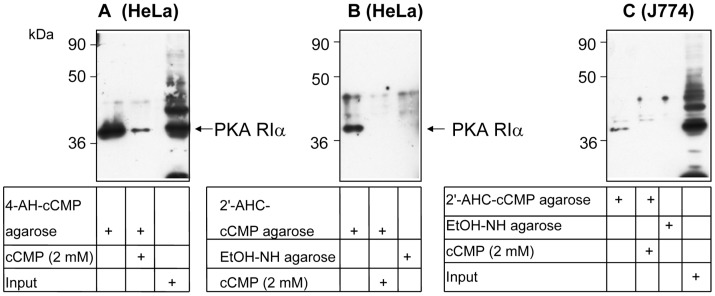
Binding of the regulatory subunit RIα of PKA to cCMP agarose. A and B, cell lysates of HeLa cells were incubated with 2′-AHC-cCMP agarose, 4-AH-cCMP agarose or EtOH-NH agarose (control agarose). In competition experiments, cCMP (2 mM) was added to cCMP agarose samples. Input designates cell lysate before incubation with agarose. C, cell lysates of HeLa cells were incubated with 2′-AHC-cCMP agarose or control agarose. RIα was detected by immunoblotting with an antibody. Numbers at the left margins of immunoblots designate markers of molecular mass standards. Representative immunoblots are shown. A and B were from the same experiment, different exposures were shown. Similar data were obtained in three independent experiments.

### Cell Culture

B103 rat neuroblastoma cells (kindly provided by Dr. E. Zoref-Shani,, Tel-Aviv, Israel) [16] were cultured in MEM RAA medium supplemented with 10% (v/v) fetal bovine serum at 37°C and 5% (v/v) CO_2_. Human HeLa cervix carcinoma cells were obtained from the American Type Culture Collection and were cultured in DMEM medium supplemented with 10% (v/v) fetal bovine serum at 37°C and 5% (v/v) CO_2_. Human HEK293 embryonic kidney cells were from the American Type Culture Collection and were cultured in DMEM medium supplemented with 10% (v/v) fetal bovine serum, non-essential amino acids and sodium pyruvate at 37°C and 5% (v/v) CO_2_. HL-60 human promyelocytic leukemia cells (kindly provided by Dr. P. Gierschik, Ulm, Germany) [17] were cultured in RPMI 1640 medium supplemented with 10% (v/v) horse bovine serum, non-essential amino acids and sodium pyruvate at 37°C and 5% (v/v) CO_2_. J774 mouse macrophages [18] were obtained from Dr. I. Just, Hannover, Germany and were cultured in DMEM medium supplemented with 10% (v/v) fetal bovine serum and 2 mM L-glutamine at 37°C and 5% (v/v) CO_2_.

**Figure 3:**
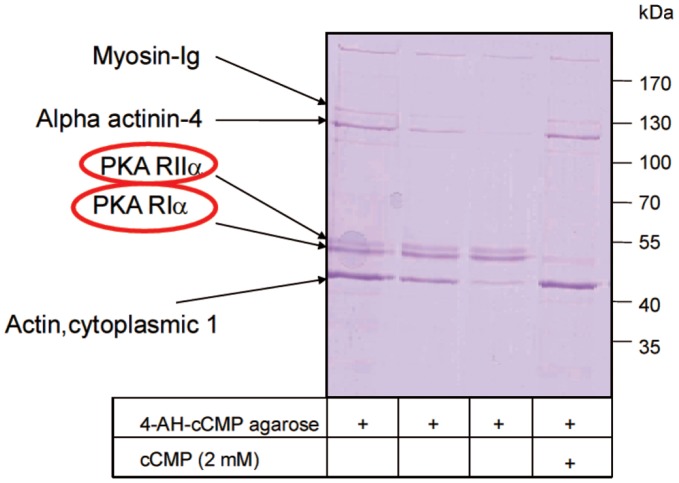
Analysis of cell lysates of HL-60 cells by gel electrophoresis and Coomassie Blue staining following incubation with 4-AH-cCMP agarose. The highly abundant proteins myosin-Ig, α-actinin-4 and cytoplasmic actin 1 bound to the 4-AH-cCMP agarose matrix non-specifically. Proteins in the ∼45 kDa region represent RIα (43 kDa) and RIIα (46 kDa), respectively, and bound to the matrix specifically since competition with cCMP (2 mM) eliminated these bands from the gel. Numbers at the right margin of the gel designate markers of molecular mass standards. After photography, the gel was cut into small pieces, and proteins were identified by MALDI and LC-MALDI mass spectrometry.

**Figure 4:**
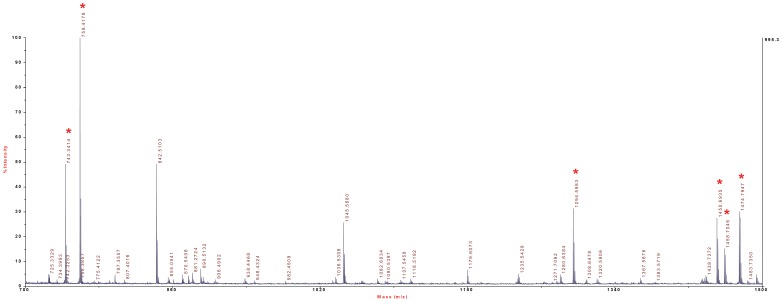
MALDI-MS spectrum of RIα peptide precursors from cell lysates of HEK293 cells. Peptides of the 43 kDa region of gels were digested and analyzed by MALDI-MS. A detailed analysis of the peptides is shown in Table 2. Peaks labelled by asterisk were subjected to MS/MS analysis.

**Figure 5:**
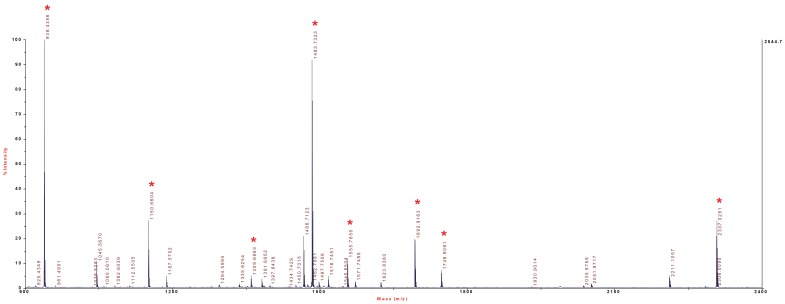
MALDI-MS spectrum of RIIα peptide precursors from cell lysates of HEK293 cells. Peptides of the 46 kDa region of gels were digested and analyzed by MALDI-MS. A detailed analysis of the peptides is shown in Table 3. Peaks labelled by asterisk were subjected to MS/MS analysis.

**Table 1:** Identification of RIα and RIIα by MALDI-MS/MS: Summary.

Cell line	Accession no.	Protein name	MW (kDa)	Mascot Score(combined, best hit)	Identified peptides	Sequence coverage
HeLa	P10644	RIα	42.955	182	6	13%
HeLa	P13861	RIIα	45.490	80	4	14%
HEK293	P10644	RIα	42.955	170	6	12%
HEK293	P13861	RIIα	45.490	403	8	27%
HL-60	P10644	RIα	42.955	247	6	16%
HL-60	P13861	RIIα	45.490	272	5	18%
B103	P09456	RIα	43.068	428	9	22%
B103	P12368	RIIα	45.512	209	3	9%

Various cell types were cultured, harvested, lyzed and analyzed by gel electrophoresis (see Figure 3). Gels were cut into small pieces and subsequently analyzed by MALDI-MS/MS. Figures 4 and 5 show representative MS spectra for peptide precursors from HEK cells, and Tables 2 and 3 provide details for the analysis of the spectra including amino acid sequences of identified peptides for RIα and RIIα.

**Table 2:** MS/MS analysis results of the peptide precursors shown in Figure 4.

RIα					
Observed	Mr(expt)	Mr(calc)	ppm	Score	Peptide
743.3401	742.3328	742.3286	5.70	35	R.EYFER.L
759.4178	758.4105	758.4075	3.95	41	K.LWGIDR.D
1294.5984	1293.5911	1293.5837	5.74	100	R.SENEEFVEVGR.L
1450.6926	1449.6854	1449.6848	0.38	59	R.RSENEEFVEVGR.L
1458.7026	1457.6954	1457.6899	3.75	27	K.NVLFSHLDDNER.S
1474.7948	1473.7875	1473.7827	3.26	53	K.VSILESLDKWER.L

Sequence coverage 12%, Mascot score 170, 6 matched queries. Data analysis from Figure 4.

**Table 3:** MS/MS analysis results of the peptide precursors shown in Figure 5.

RIIα					
Observed	Mr(expt)	Mr(calc)	ppm	Score	Peptide
938.4357	937.4285	937.4254	3.31	58	R.SVGQYDNR.G
1150.6609	1149.6536	1149.6506	2.63	72	R.APASVLPAATPR.Q
1359.6845	1358.6772	1358.6830	−4.29	55	R.NISHYEEQLVK.M
1483.7328	1482.7255	1482.7215	2.68	108	R.QSLGHPPPEPGPDR.V
1555.7653	1554.7580	1554.7500	5.11	32	R.GSFGELALMYNTPR.A
1692.9164	1691.9091	1691.8995	5.68	85	K.GQYFGELALVTNKPR.A
1746.9063	1745.8990	1745.8948	2.39	27	R.AATIVATSEGSLWGLDR.V
2307.0256	2306.0184	2306.0087	4.17	170	K.ADEHVIDQGDDGDNFYVIER.G

Sequence coverage 27%, Mascot score 403, 8 matched queries. Data analysis from Figure 5.

### cCMP Agarose Affinity Chromatography

**Table 4:** Identification of RIα and RIIα by LC-MALDI-MS/MS in B103 cells.

*Accession no.*	*MW (Da)*	*Protein name*			
P09456	43068	cAMP-dependent protein kinase type Iα regulatory subunit			
*Observed*	*Mr(expt)*	*Mr(calc)*	*ppm*	*Score*	*Peptide*
721.4604	720.4531	720.3840	96.0	45	K.TMAALAK.A + Oxidation (M)
743.3256	742.3183	742.3286	−13.79	27	R.EYFER.L
759.4175	758.4103	758.4075	3.62	34	K.LWGIDR.D
844.5325	843.5252	843.4814	51.9	37	R.QIQSLQK.S
890.5557	889.5485	889.5055	48.3	50	R.ILMGSTLR.K
936.6090	935.6017	935.5552	49.7	55	K.HNIQALLK.D
1046.5131	1045.5058	1045.4790	25.6	63	K.MYEEFLSK.V
1174.6108	1173.6036	1173.5740	25.2	60	R.KMYEEFLSK.V
1271.7720	1270.7647	1270.7067	45.6	61	R.VLGPCSDILKR.N + Propionamide (C)
1294.6943	1293.6871	1293.5837	79.9	104	R.SENEEFVEVGR.L
1438.8260	1437.8188	1437.7286	62.7	57	R.SLRECELYVQK.H + Propionamide (C)
1450.8023	1449.7950	1449.6848	76.0	91	R.RSENEEFVEVGR.L
1458.7551	1457.7479	1457.6899	39.8	94	K.NVLFSHLDDNER.S
1474.7238	1473.7165	1473.7827	−44.95	35	K.VSILESLDKWER.L
1859.8817	1858.8744	1858.9313	−30.57	129	R.LTVADALEPVQFEDGQK.I
1930.8989	1929.8917	1929.8956	−2.03	159	R.GAISAEVYTEEDAASYVR.K
1977.1696	1976.1623	1975.9851	89.7	69	R.TDSREDEISPPPPNPVVK.G
2059.0046	2057.9974	2057.9905	3.32	107	R.GAISAEVYTEEDAASYVRK.V
2087.0054	2085.9981	2085.9967	0.68	38	R.RGAISAEVYTEEDAASYVR.K
**P12368**	**45512**	**cAMP-dependent protein kinase type IIα regulatory subunit**			
***Observed***	***Mr(expt)***	***Mr(calc)***	***ppm***	***Score***	***Peptide***
938.4172	937.4100	937.4254	−16.42	51	R.SVGQYDNR.G
1051.6035	1050.5962	1050.5346	58.7	77	R.AASAYAVGDVK.C
1359.6910	1358.6838	1358.6830	0.56	34	R.NISHYEEQLVK.M
1571.6670	1570.6597	1570.7450	−54.27	50	R.GSFGELALMYNTPR.A + Oxidation (M)
1623.8324	1622.8251	1622.8264	−0.79	70	R.GTYDILVTKDNQTR.S
1692.8108	1691.8035	1691.8995	−56.73	82	K.GQYFGELALVTNKPR.A
1732.7730	1731.7657	1731.8792	−65.53	49	R.AATIVATSDGSLWGLDR.V
2336.8967	2335.8895	2336.0193	−55.59	125	K.TDEHVIDQGDDGDNFYVIER.G
2677.1609	2676.1536	2676.2668	−42.28	44	K.IVKTDEHVIDQGDDGDNFYVIER.G

**Table 5:** Identification of RIα and RIIα by LC-MALDI-MS/MS in HEK293 cells.

*Accession no.*	*MW (Da)*	*Protein name*			
P10644	42955	cAMP-dependent protein kinase type Iα regulatory subunit			
*Observed*	*Mr (expt)*	*Mr (calc)*	*Ppm*	*Score*	*Peptide*
906.5111	905.5038	905.5004	3.76	35	R.ILMGSTLR.K + Oxidation (M)
1294.6105	1293.6032	1293.5837	15.1	100	R.SENEEFVEVGR.L
1450.6728	1449.6656	1449.6848	−13.26	91	R.RSENEEFVEVGR.L
1458.7145	1457.7072	1457.6899	11.9	83	K.NVLFSHLDDNER.S
1930.8883	1929.8810	1929.8956	−7.53	131	R.GAISAEVYTEEDAASYVR.K
**P13861**	**45490**	**cAMP-dependent protein kinase type IIα regulatory subunit**			
***Observed***	***Mr(expt)***	***Mr(calc)***	***Ppm***	***Score***	***Peptide***
1150.6508	1149.6435	1149.6506	−6.18	84	R.APASVLPAATPR.Q
1187.5758	1186.5685	1186.5578	9.03	103	K.DGGNQEVEIAR.C
1359.7057	1358.6984	1358.6830	11.3	58	R.NISHYEEQLVK.M
1483.7291	1482.7219	1482.7215	0.21	76	R.QSLGHPPPEPGPDR.V
1516.7294	1515.7221	1515.7277	−3.71	98	K.SNKDGGNQEVEIAR.C
1571.7419	1570.7347	1570.7450	−6.56	63	R.GSFGELALMYNTPR.A + Oxidation (M)
1692.9087	1691.9014	1691.8995	1.14	95	K.GQYFGELALVTNKPR.A
2307.0486	2306.0413	2306.0087	14.1	158	K.ADEHVIDQGDDGDNFYVIER.G

**Table 6:** Identification of RIα and RIIα by LC-MALDI-MS/MS in HL-60 cells.

*Accession no.*	*MW (Da)*	*Protein name*			
P10644	42955	cAMP-dependent protein kinase type Iα regulatory subunit			
*Observed*	*Mr(expt)*	*Mr(calc)*	*Ppm*	*Score*	*Peptide*
743.3398	742.3326	742.3286	5.36	26	R.EYFER.L
1062.4757	1061.4684	1061.4739	−5.17	29	K.MYEEFLSK.V + Oxidation (M)
1294.6033	1293.5960	1293.5837	9.51	91	R.SENEEFVEVGR.L
1450.7048	1449.6976	1449.6848	8.80	100	R.RSENEEFVEVGR.L
1458.7072	1457.6999	1457.6899	6.85	95	K.NVLFSHLDDNER.S
1859.9345	1858.9272	1858.9313	−2.20	98	R.LTVADALEPVQFEDGQK.I
1976.9853	1975.9781	1975.9851	−3.54	55	R.TDSREDEISPPPPNPVVK.G
**P13861**	**45490**	**cAMP-dependent protein kinase type IIα regulatory subunit**			
***Observed***	***Mr(expt)***	***Mr(calc)***	***ppm***	***Score***	***Peptide***
938.4013	937.3940	937.4254	−33.41	40	R.SVGQYDNR.G
1187.4938	1186.4865	1186.5578	−60.11	75	K.DGGNQEVEIAR.C
1359.6887	1358.6814	1358.6830	−1.14	46	R.NISHYEEQLVK.M
1483.6348	1482.6275	1482.7215	−63.43	56	R.QSLGHPPPEPGPDR.V
2307.0242	2306.0169	2306.0087	3.53	68	K.ADEHVIDQGDDGDNFYVIER.G

Cells were harvested and suspended in lysis buffer consisting of 40 mM β-glycerolphosphate, 100 mM NaF, 4 mM Na_3_VO_4_, 2% (m/v) Triton X-100, 100 mM NaCl, 60 mM NaPP_i_ and 20 mM Tris/HCl, pH 7.5. Protein concentration was determined using the BCA protein assay. 2′-AHC-cCMP agarose, 4-AH-cCMP agarose and EtOH-NH agarose (30 µl each) were equilibrated three times with wash buffer consisting of 1 mM dithiothreitol, 1% (m/v) Triton X-100, 1 mM Na_3_VO_4_, 50 mM NaF, 154 mM NaCl and 20 mM Tris/HCl, pH 7.5. Agarose beads were incubated with 2 mg of cell lysate protein in wash buffer (total volume 500 µl) in the presence of 100 µM isobutyl-methylxanthine under rotation at 30 rpm at 4°C overnight. In order to detect non-specific binding, 2 mM cCMP was included in some samples. Samples were then centrifuged at 1,000 g for 3 min at 4°C, and beads were washed three times with 500 µl of wash buffer, followed by addition of 25 µl of 2× sample buffer. Samples were heated for 10 min at 95°C. For alkylation of cysteine residues 1 µL of an acrylamide solution (40%, m/v) was added and incubated at room temperature for 30 min. Proteins were subsequently separated by sodium dodecyl sulfate gel electrophoresis in gels containing 10% (m/v) acrylamide.

### Immunoblotting

Gels were blotted onto nitrocellulose membranes. Membranes were incubated with anti-RIα Ig (1∶500) over night, followed by a 2 h incubation with anti-mouse IgG from sheep (1∶2,000). Bands were visualized using the Signal WestPico Luminol Enhancer and Stable Peroxidase Solution (Thermo Fisher Scientific, Rockford, IL, USA).

### Sample Preparation for MS Analysis

Following photography for documentation, protein-containing gel lanes were cut into small pieces and destained with ACN (50%, v/v) in 20 mM NH_4_HCO_3_. Subsequently, ACN (100%) was added until gel pieces were dry and ACN was removed in a vacuum centrifuge. Trypsin was added at a concentration of 10 ng/µL in 20 mM NH_4_CO_3_ and 10% (v/v) ACN and the protein digest was performed at 37°C over night. Peptides were extracted by incubation of samples with 50 µl of 10% (v/v) ACN and 0.5% (v/v) trifluoroacetic acid (TFA) at room temperature and shaking at 300 rpm for 30 min. The supernatant fluid was transferred into a new vial, and the extraction was repeated twice using increasing concentrations of ACN (30%, 50%). Following vacuum drying, samples were dissolved in 5 µl of 5% (v/v) ACN and 0.2% (v/v) TFA for matrix-assisted laser desorption/ionization (MALDI)-MS analysis. Samples (0.5 µl) were spotted onto a MALDI target plate (AB Sciex, Darmstadt, Germany) and mixed with 0.8 µl α-cyano-4-hydroxycinnamic acid (CHCA) (4 mg/mL in 50% ACN, 0.2% TFA) using the dried droplet method.

### LC Analysis

Peptide separation was performed by reversed phase chromatography using a nano-LC system (Dionex, Idstein, Germany) which consists of an autosampler (Famos), a loading pump (Switchos), a gradient pump (Ultimate) and a microfraction collector (Probot). An aliquot of up to 20 µL of each sample was injected onto a C18 trap column (PepMap 300 µm×5 mm, 3 µm, 100 Å, Dionex) with 2% (v/v) acetonitrile (ACN) in 0.1% (v/v) TFA and a flow rate of 30 µL/min. Peptides were eluted onto a separation column (PepMap, C18 reversed phase material, 75 µm×150 mm, 3 µm, 100 Å, Dionex) and separated using eluent A with 5% (v/v) acetonitrile in 0.1% (v/v) TFA and eluent B with 80% (v/v) acetonitrile in 0.1% (v/v) TFA with a gradient from 10% to 40% eluent B in 134 min and 40% to 100% eluent B in 10 min. Samples were spotted directly onto a MALDI target plate (AB Sciex) that had been prespotted with CHCA matrix as described above. A sheath liquid of 50% (v/v) ACN was applied and subsequently spots were recrystallized using 50% (v/v) ACN and 0.1% (v/v) TFA.

### MALDI-MS/MS and Protein Identification

Samples were analyzed by MALDI-MS using the (time-of-flight/ time-of-flight) TOF/TOF 5800 mass spectrometer (AB Sciex). MS spectra were calibrated using external calibration with a peptide standard (AB Sciex). For internal calibration peptides with m/z values of 842.51 and 2211.103 descending from trypsin were used. MS/MS calibration was performed using fragments of the angiotensin peptide m/z 1296.685 present in the peptide standard. Initially, samples were measured in MS mode. The 30 most intense peaks were selected for fragmentation and MS/MS-analysis. MS spectra were searched against the SwissProt/Uniprot database using the Mascot search engine version 2.2.04 (Matrix Science, London, UK) and the results were processed with Protein Pilot software 3.0 (AB Sciex). Error tolerance was set to 100 ppm for precursor masses and 0.3 Da for fragment masses. Methionine oxidation and cysteine alkylation by propionamide were used as modifications. Proteins were considered identified if at least two peptides with a peptide ion score of each ≥ 25 each were identified.

## Results

### Identification of PKA RIα by Immunoblotting

The cNMP agarose affinity approach has already been proven to be successful at identifying cNMP-binding proteins [12], [15]. PKA RIα is expressed in many cell types [1]. We probed both 2′-AHC-cCMP agarose and 4-AH-cCMP agarose in HeLa cells, a widely used cell culture model (Figure 2A and 2B). Both matrices bound RIα as assessed by immunoblotting. Binding was specific since cCMP strongly inhibited RIα binding to cCMP matrices, and the control agarose devoid of the cCMP moiety did not bind RIα. In J774 mouse macrophages, 2′-AHC-cCMP agarose also bound RIα in a specific manner as assessed by the use of cCMP as competing ligand and control agarose (Figure 2C). 4-AH-cCMP agarose was more effective than 2′-AHC agarose at binding RIα (compare Figure 2A versus Figure 2B and 2C). Therefore, all further experiments were performed with 4-AH-cCMP agarose.

### Identification of RIα and RIIα by MALDI-MS/MS

Figure S1 shows the sequence alignment of human RIα and RIIα. The sequence identity between the two isoforms amounts to 38%, but the amino acid sequences are sufficiently different from each other to allow for unequivocal protein identification by peptide analysis via MALDI-MS/MS. Figure 3 shows the Coomassie Blue-stained gel of cell lysates of HL-60 cells following incubation with 4-AH-cCMP agarose. The gel shows two bands in the ∼45 kDa region that were competed for by cCMP. The gel was cut into thin slices, proteins were digested and peptides were analyzed by MALDI-MS/MS. This analysis showed that highly abundant proteins, i.e. myosin-Ig, α-actinin-4 and cytoplasmic actin bound non-specifically to 4-AH-cCMP agarose, i.e. the binding of these proteins was not competed for by cCMP (Figure 3). In contrast, the bands in the ∼45 kDa region competed for by cCMP were identified as RIα and RIIα. Figure 4 and 5 show representative peptide precursor MS spectra for RIα and RIIα from HEK293 cells, respectively. Table 1 provides a summary of the MALDI-MS/MS analysis of the ∼45 kDa region of HeLa cells, HEK293 cells, HL-60 cells and B103 cells. In all four cell types, RIα and RIIα were identified with sequence coverages ranging from 9–27%, the number of identified peptides ranging from 3–9 and highly significant combined Mascot score ranging from 80–428. Tables 2 and 3 list the amino acid sequences of peptides analyzed in Figures 4 and 5.

We further refined the analysis of proteins bound to 4-AH-cCMP agarose by separating peptides of the 45 kDa region using reversed phase chromatography prior to MALDI-MS/MS (LC-MALDI). Tables 4, 5, 6 show that in this analysis, RIα and RIIα were unequivocally identified in B103 cells, HEK293 cells and HL-60 cells, the number of identified peptides ranged from 5–19 and peptide ion scores of individual peptides ranged from 26–159.

## Discussion

For many years, research on cCMP barely progressed because of non-reproducible results [3], [4] technical difficulties in determination of the activity of cCMP-forming enzymes [5], [6] lack of sufficiently sensitive and specific cCMP detection techniques and absence of experimental tools to detect cCMP-binding proteins [3]. Recently, we could unequivocally demonstrate that certain bacterial adenylyl cyclase toxins also produce cCMP [9] and recombinant soluble guanylyl cyclase α_1_β_1_ does so, too [10]. Moreover, we showed that the recombinant regulatory subunits RIα and RIIα of PKA bind cCMP, resulting in dissociation of the R subunits from the catalytic subunits and subsequent protein phosphorylation [11]. Thus, a functional effect of cCMP on clearly defined proteins was finally shown.

Considering the success of the cNMP agarose approach to identify cNMP-binding proteins [12], [15] the recent results on cCMP synthesis and cCMP effects on PKA prompted us to synthesize and test two cCMP agaroses (Figure 1) in order to identify cCMP-binding proteins. The application of both cCMP agaroses was straightforward, EtOH-NH agarose and competition with cCMP serving as specificity control (Figure 2 and 3). In immunoblotting experiments we detected RIα (Figure 2). In MALDI-MS/MS analysis, a traditional approach analyzing gel slices (Figure 3, 4 and 5 and Tables 1, 2, 3) and in a more advanced approach applying additional reversed phase chromatography prior to MS analysis (Tables 4, 5, 6), we unequivocally identified RIα and RIIα in several cell types as proteins specifically binding to 4-AH-cCMP agarose.

We were somewhat surprised that the cCMP-agarose approach worked so well considering the fact that cCMP is only a low-potency activator of PKA [11]. RIα appears to possess considerable conformational flexibility since the attachment of the affinity ligand to the matrix, either via the 2′-O-ribosyl group or the 4-NH group of the pyrimidine base worked. The higher efficacy of 4-AH-cCMP agarose compared to 2′-AHC-cCMP agarose at binding RIα can be explained by the fact that the 2′-OH group of cNMPs is important for interaction with the protein [19]. Thus, our data provide a compelling example for the notion that low-affinity interactions between a protein and a ligand cannot necessarily be dismissed as non-specific. Exceedingly high affinity of a protein to a ligand may impede with subsequent dissociation of the protein from the affinity matrix [12], [15]. Evidently, in cCMP agarose matrices, steric ligand accessibility and the balance between sufficient binding affinity and subsequent protein elution are quite right. In intact cells, cCMP, due to its stability (see discussion below) [8] may accumulate in specific PKA-containing cell compartments so that sufficiently high cCMP concentrations for PKA activation build up. In fact, in a recent study, we have shown that in certain cells, overall cCMP concentrations are in the range of ∼30 pmol/10^6^ cells which is just three-fold lower than the corresponding cAMP concentration [20].

In previous studies we showed that cCMP induces vasodilatation and inhibition of platelet aggregation via cGMP-dependent protein kinase (PKG) and that cCMP also binds to purified PKG [11], [21]. However, in none of the cell types studied here and with none of the experimental approaches did we identify PKG as protein binding to cCMP agarose. This apparent discrepancy may be due to the fact that the expression of PKG is too low in the cell types studied. As a consequence, binding of PKG to cCMP agarose may be below the detection limit of the currently available mass spectrometers. Thus, in future studies, PKG-enriched cells such as platelets and smooth muscle cells will have to be examined. Alternatively or additionally, there may be steric conflicts in the binding of PKG to the two cCMP agarose matrices. A hint towards steric problems may be the fact that in contrast to the situation with PKA, cCMP is only a partial activator of PKG [11]. Accordingly, it will be necessary to develop affinity matrices with different ligand densities, space lengths between the agarose and the cNMP and different attachment positions of the cNMP to the linker. Figure 1 illustrates some of the chemical possibilities to optimize affinity matrices.

It is also noteworthy that our studies did not identify cNMP-degrading phosphodiesterases as target proteins for cCMP. Previous studies claimed the existence of a specific cCMP-degrading phosphodiesterase [7] but its molecular identity remained elusive. Rather, in a recent study, we examined a broad panel of human phosphodiesterases and found none of them to cleave cCMP [8]. Our negative cCMP affinity matrix data regarding phosphodiesterases fit to the functional data. These data raise the question through which mechanism cCMP is inactivated if it is, indeed, a second messenger. Transmembrane export may be an inactivation mechanism but the affinity of the interaction of such transporters with cCMP may be too low to be detected by our affinity ligand approach [22], [23]. In fact, transporters of the MRP family accept structurally very diverse substrates so that a specific interaction with an affinity ligand cannot necessarily be expected [23]. Lastly, in our study, we did neither detect Epac nor cNMP-regulated ion channels as cCMP-binding proteins [24], [25]. As is the case for PKG and phosphodiesterases, such negative data do not exclude the existence of other cCMP-binding proteins. These proteins may simply have gone unnoticed in our analysis for various technical reasons including suitability of affinity matrices and sensitivity of MS detection methods.

In conclusion, in this study we provided proof of principle that the use of cCMP affinity matrices is a useful approach to identify cCMP-binding proteins. We anticipate that the systematic application of this approach in terms of the development of multiple matrices and the analysis of multiple cell types, together with refined LC-MS techniques, will lead to the identification of additional cCMP-binding proteins, some of which may turn out to be specific for cCMP.

## Supporting Information

Figure S1 **Sequence comparison of RIα and RIIα.** Amino acid sequences of human RIα and RIIα were aligned, using the one-letter code. Sequences were aligned in http://www.uniprot.org/blast/. Sequence identity amounts to 38%.
(JPG)
