# Supplementary figures and images for: Binding of Regulatory Subunits of Cyclic AMP-Dependent Protein Kinase to Cyclic CMP Agarose
Source: PLoS One. 2012 Jul 9;7(7):e39848. doi: 10.1371/journal.pone.0039848 (PMC3392273; doi:10.1371/journal.pone.0039848)

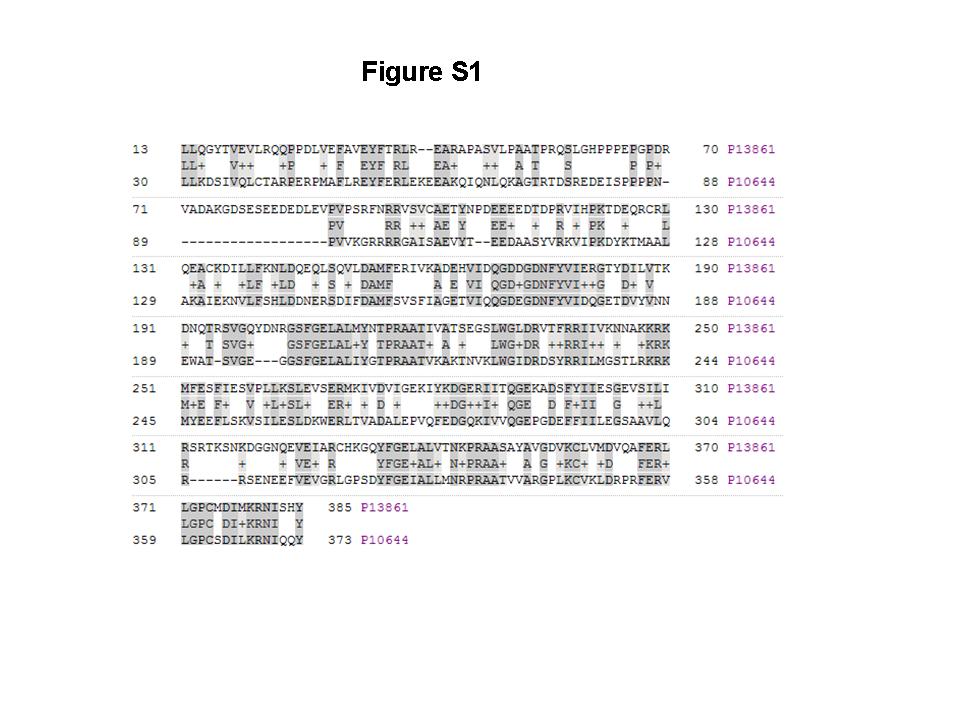

Supplement: Figure S1 — Sequence comparison of RIα and RIIα. Amino acid sequences of human RIα and RIIα were aligned, using the one-letter code. Sequences were aligned in http://www.uniprot.org/blast/. Sequence identity amounts to 38%. (JPG) [file pone.0039848.s001.jpg]
